# Factors associated with carbon dioxide transfer in an experimental model of severe acute kidney injury and hypoventilation during high bicarbonate continuous renal replacement therapy and oxygenation membrane support

**DOI:** 10.62675/2965-2774.20240005-en

**Published:** 2024-06-19

**Authors:** Yuri de Albuquerque Pessoa dos Santos, Luis Carlos Maia Cardozo, Pedro Vitale Mendes, Bruno Adler Maccagnan Pinheiro Besen, Marcelo Park

**Affiliations:** 1 Hospital das Clínicas Faculdade de Medicina Universidade de São Paulo São Paulo SP Brazil Medical Intensive Care Unit, Hospital das Clínicas, Faculdade de Medicina, Universidade de São Paulo - São Paulo (SP), Brazil.

**Keywords:** Carbon dioxide, Bicarbonates, Respiratory insufficiency, Acute kidney injury, Renal replacement therapy, Animal

## Abstract

**Objective:**

To investigate the factors influencing carbon dioxide transfer in a system that integrates an oxygenation membrane in series with high-bicarbonate continuous veno-venous hemodialysis in hypercapnic animals.

**Methods:**

In an experimental setting, we induced severe acute kidney injury and hypercapnia in five female Landrace pigs. Subsequently, we initiated high (40mEq/L) bicarbonate continuous veno-venous hemodialysis with an oxygenation membrane in series to maintain a pH above 7.25. At intervals of 1 hour, 6 hours, and 12 hours following the initiation of continuous veno-venous hemodialysis, we performed standardized sweep gas flow titration to quantify carbon dioxide transfer. We evaluated factors associated with carbon dioxide transfer through the membrane lung with a mixed linear model.

**Results:**

A total of 20 sweep gas flow titration procedures were conducted, yielding 84 measurements of carbon dioxide transfer. Multivariate analysis revealed associations among the following (coefficients ± standard errors): core temperature (+7.8 ± 1.6 °C, p < 0.001), premembrane partial pressure of carbon dioxide (+0.2 ± 0.1/mmHg, p < 0.001), hemoglobin level (+3.5 ± 0.6/g/dL, p < 0.001), sweep gas flow (+6.2 ± 0.2/L/minute, p < 0.001), and arterial oxygen saturation (-0.5 ± 0.2%, p = 0.019). Among these variables, and within the physiological ranges evaluated, sweep gas flow was the primary modifiable factor influencing the efficacy of low-blood-flow carbon dioxide removal.

**Conclusion:**

Sweep gas flow is the main carbon dioxide removal-related variable during continuous veno-venous hemodialysis with a high bicarbonate level coupled with an oxygenator. Other carbon dioxide transfer modulating variables included the hemoglobin level, arterial oxygen saturation, partial pressure of carbon dioxide and core temperature. These results should be interpreted as exploratory to inform other well-designed experimental or clinical studies.

## INTRODUCTION

Low-flow extracorporeal circuits are effective for carbon dioxide (CO_2_) removal due to their high CO_2_ diffusibility.^(1)^ These systems have been employed as rescue therapies in clinical settings.^(2)^However, the use of smaller biocompatible oxygenation membranes (< 0.8m^2^) is insufficient for adequately correcting severe respiratory acidosis.^(3,4)^In contrast, high (40mEq/L) bicarbonate dialysates in continuous veno-venous hemodialysis (CVVHD) improve pH control in bench models of hypercapnic acute kidney injury.^(5)^The combination of small surface oxygenation membranes in series with high-bicarbonate CVVHD may be a potential intervention for patients with respiratory failure and acute kidney injury, but its efficacy has been poorly explored in bench studies.

We aimed to investigate the factors influencing CO_2_ transfer in a system that integrates an oxygenation membrane in series with high-bicarbonate CVVHD in hypercapnic animals.

## METHODS

This was a planned secondary analysis of an experiment conducted at the *Faculdade de Medicina* of the *Universidade de São Paulo*, approved by the Animal Experimentation Ethics Committee (CEUA-17699/2022). The results of the primary study were not published at the time of the publication of this manuscript.

### Instrumentation

The study prioritized animal welfare, with animals being anesthetized and instrumented as previously described.^(5)^ Following anesthesia, we placed a central venous line, a 12-French, 16cm venous dialysis catheter (Arrow™, PA, USA), a Swan-Ganz catheter (Edwards Lifesciences^TM^, Irvine, USA), and an arterial line. A median laparotomy followed by a cystostomy was performed to confirm anuria, and the renal hilum was ligated *en bloc*. The animals were stabilized for one hour after surgery.

### Hypercapnia protocol

After stabilization, we collected baseline data and induced hypercapnia by reducing the tidal volume to two-thirds while adjusting the respiratory rate to 40 breaths/minute. One hour later, we initiated CVVHD in series with an oxygenator. Over the next 12 hours, we fine-tuned the tidal volume hourly to a target arterial pH > 7.25, aiming for a minimal tidal volume of 3.5mL/kg. During this period, extracorporeal support was maintained, and clinical and laboratory data were collected hourly.

### Extracorporeal metabolic and respiratory support

We used an Fx80^®^ dialysis filter (Fresenius Kabi LTDA) with 30mL/kg of dialysate and a blood flow rate of 3 - 4mL/kg/minute. Predialysis filter heparin was administered as a 15 - 20IU/kg *bolus*, followed by an hourly infusion at the same rate. The phosphate-free dialysate composition was [Na^+^] = 140.05mEq/L, [Cl-] = 103.85mEq/L, [K^+^] = 3.81mEq/L, and [HCO_3_-] = 40.02mEq/L. The high bicarbonate dialysate aimed to optimize the metabolic component of pH^(5)^ to allow a faster reduction in tidal volume when combined with the decarboxylation effect of the oxygenator.

For decarboxylation, we used a Biocube2000 oxygenator (Nipro Medical LTDA), which features a 0.4m^2^ exchange surface of polymethylpentene fibers. The sweep gas flow (SGF) was maintained at 10L/minute using only oxygen (FdO_2_ = 100%).

### Carbon dioxide transfer measurement

We quantified CO_2_ transfer by estimating the partial pressure of CO_2_ and the volume of gas exhaled from the oxygenator’s outlet, ensuring that no gas leaked. The partial pressure of CO_2_ was estimated using an infrared end-tidal CO_2_ (E_T_CO_2_) sensor integrated into the DX 2020 multiparametric monitor (Dixtal, LTDA, São Paulo, Brazil).

The exhaled gas volume per minute was measured with a micrometrically precise adjustable flow meter connected to a Sechrist3500^®^ oxygen air blender (Sechrist Industries, INC, Anaheim, CA, USA).

Carbon dioxide transfer was defined as the proportion of exhaled gas per minute including the measured CO_2_ partial pressure, estimated as follows: CO_2_ transfer = (E_T_CO_2_/barometric pressure) × (gas volume/minute). The results are expressed in mL/minute, considering the average barometric pressure of 700mmHg in São Paulo. This methodology is consistent with the techniques employed by Theodor Kolobow^(6)^ and has been further refined and tested by our research group.^(7)^

### Sweep gas flow titration protocol

Sweep gas flow titration (SGFt) was conducted using predefined SGF levels ranging from 0 to 10L/minute, a micro/macrometric oxygen precision flowmeter and a flow regulator (Prevtech, São Paulo, SP, Brazil). For each SGF measurement, the flow was reduced from an initial 10L/minute to the specified level. We observed the E_T_CO_2_ curve and value until stabilization for 10 seconds, at which point the E_T_CO_2_ was recorded as the equilibrated exhaled CO_2_ partial pressure at that SGF. In cases where the E_T_CO_2_ was undetectable at a given SGF, the previous CO_2_ partial pressure was considered the trough, and CO_2_ transfer was considered the plateau.

The SGFt was prespecified and conducted at 1 - 6 - 12 hours for all animals, with additional measurements taken as needed.

### Statistical analysis

Clinical data are presented as medians [25^th^- 75^th^ percentiles]. The associations of SGF and CO_2_ transfer with other potential influencing factors are presented using spaghetti and spider plots, respectively. Using linear mixed models with each animal as a random factor to account for clustered observations, we analyzed measurements over time and the multivariable association of potential independent factors with CO_2_ transfer, employing backward elimination for the latter. These factors, drawn from prior literature,^(6-10)^included premembrane CO_2_ partial pressure (PCO_2_), hemoglobin levels, arterial oxygen saturation (SaO_2_), SGF, and core temperature,^(6-10)^ with PaCO_2_ serving as a surrogate for premembrane PCO_2_. Blood flow, an independent factor in extracorporeal membrane oxygenation (ECMO) studies with higher flow variations,^(7,9)^ was excluded from the multivariable analysis of CVVHD due to low flow rates. Statistical analysis was performed with R.^(11)^

## RESULTS

We included five animals with an average weight of 33.1kg (28.7 - 35.0kg), and 20 SGFt procedures and 84 CO_2_ transfer measurements were performed. Hemodynamic, respiratory, and metabolic characteristics before SGFt are detailed in table 1. Tidal volume decreased alongside a significant increase in PaCO_2_, although the pH remained above 7.25.

**Table 1:** Respiratory, hemodynamic and metabolic physiological variables just before the membrane sweep gas flow titration

Timepoints	Baseline	1 hour	2 hours	3 hours	4 hours	6 hours	7 hours	12 hours	p value*
VCO_2_†/animals sample	84/5	84/5	37/5	38/5	12/5	38/5	42/5	25/5	
Respiratory variables									
Tidal volume (mL)	300 [260,320]	200 [200,220]	180 [180,220]	170 [160,200]	160 [160,160]	150 [140,160]	140 [120,180]	120 [120,180]	< 0.010
Respiratory rate (bpm)	35 [25,38]	40 [25,40]	40 [25,40]	40 [25,40]	40 [40,40]	40 [40,40]	40 [40,40]	40 [40,40]	< 0.010
FiO_2_ (%)	21 [21,21]	25 [25,31]	25 [21,28]	30 [25,35]	25 [25,25]	30 [25,30]	35 [35,40]	38 [30,38]	< 0.010
EtCO_2_ (mmHg)	36 [30,37]	38.50 [36,46]	43 [39,56]	47 [38,67]	43 [43,43]	47 [45,49]	62 [41,64]	87 [42,87]	< 0.010
Hemodynamic variables									
Cardiac output (L/minute)	3.0 [2.6,3.6]	3.3 [2.4,3.4]	3.1 [3.0,3.5]	3.3 [2.5,3.5]	3.4 [3.4,3.4]	4.3 [3.1,5.6]	4.5 [4.0,8.4]	4.9 [4.0,4.9]	< 0.001
Heart rate (beats/minute)	111 [80,159]	157 [84,157]	129 [93,146]	137 [87,195]	132 [132,132]	157 [140,187]	154 [121,209]	129 [129,196]	< 0.010
PAPm (mmHg)	26 [25,35]	27 [25,28]	28 [26,28]	26 [23,27]	27 [27,27]	30 [29,35]	27 [27,32]	28 [28,30]	< 0.010
APm (mmHg)	121 [101,123]	101.50 [80,117]	91 [88,94]	84 [70,86]	89 [89,89]	84 [71,90]	78 [75,105]	71 [41,71]	< 0.010
CVP (mmHg)	8 [7,10]	5 [5,9]	5 [3,7]	6 [3,7]	7 [7,7]	6 [3,7]	8 [7,9]	8 [8,8]	< 0.010
PAOP (mmHg)	12 [9,15]	12 [9,12]	8 [7,10]	9 [6,12]	10 [10,10]	9 [7,9]	9 [9,12]	8 [8,10]	< 0.010
Metabolic and CRRT variables									
Core temperature (°C)	38.5 [38.3,39.1]	37.8 [37.1,37.8]	37.7 [37.1,38.2]	38.2 [37.0,38.4]	38.4 [38.4,38.4]	37.8 [37.0,38.7]	38.1 [37.8,39.0]	38.0 [37.6,38.0]	< 0.001
Blood flow (mL/minute)	187.50 [96,202]	187.50 [96,204]	167 [96,205]	172 [95,182]	208 [208,208]	178 [96,205]	182 [96,198]	96 [96,210]	< 0.010
Hemoglobin level (g/dL)	9.8 [7.8,11.0]	10.0 [8.4,11.0]	10.2 [7.5,11.1]	10.2 [7.5,11.1]	7.7 [7.7,7.7]	11.0 [10.7,11.3]	8.5 [8.2,11.4]	11.1 [7.5,11.1]	< 0.001
pH	7.44 [7.41,7.49]	7.36 [7.31,7.42]	7.39 [7.30,7.42]	7.39 [7.30,7.42]	7.36 [7.36,7.36]	7.32 [7.31,7.37]	7.26 [7.22,7.33]	7.25 [7.25,7.32]	< 0.001
PaCO_2_ (mmHg)	31 [30,37.90]	36 [35,48.15]	43 [37,58.40]	43 [37,58.40]	44 [44,44]	50 [50,51]	64.20 [56,67.90]	86.70 [43,86.70]	< 0.010
PaO_2_ (mmHg)	75.90 [70,86]	76.47 [73.95,85]	75 [72,76]	75 [72,76]	77 [77,77]	83 [63,91]	89.30 [73,91]	80.90 [80.90,92]	< 0.010
Oxygen saturation (%)	97 [93.20,98]	95.50 [90.55,99.15]	93 [87.90,94.50]	93 [87.90,94.50]	93.80 [93.80,93.80]	95 [94.60,96.20]	88 [84,89.30]	90.30 [90.30,95]	< 0.010
SBE (mEq/L)	-0.2 [-1.4,0.2]	1.1 [-0.8,3.9]	1.6 [0.1,2.4]	1.6 [0.1,2.4]	0.3 [0.3,0.3]	-0.2 [-4.6,4.2]	0.1 [-1.7,7.4]	9.3 [-3.3,9.3]	< 0.001

VCO_2_ - *carbon dioxide production*; bpm - breaths per minute; FiO_2_ - inspiratory oxygen fraction; EtCO_2_ - end-tidal *carbon dioxide* partial pressure; PAPm - mean pulmonary arterial pressure; APm - systemic mean arterial pressure; CVP - central venous pressure; PAOP - pulmonary occlusion arterial pressure; CRRT - continuous renal replacement therapy; PCO_2_ - partial pressure of carbon dioxide; PaO_2_ - partial pressure of oxygen; SBE - standard base excess. * p value extracted from the time evolution of each variable, using a mixed linear model factor *versus* time interaction with the individual animal as the random factor; † Here is the number of sweep gas flow titration sequences.

The multivariable analysis yielded the following results [coefficient ± standard error (p value)]: an intercept = -271.6 ± 63.4 (p < 0.001), a core temperature (ºC) = +7.8 ± 1.6 (p < 0.001), a premembrane PCO_2_ (mmHg) = +0.2 ± 0.1 (p < 0.001), a hemoglobin level (g/dL) = +3.5 ± 0.6 (p < 0.001), an SaO_2_ (%) = -0.5 ± 0.2 (p = 0.019), and an SGF (L/minute) = +6.2 ± 0.2 (p < 0.001).

**Figure 1:**
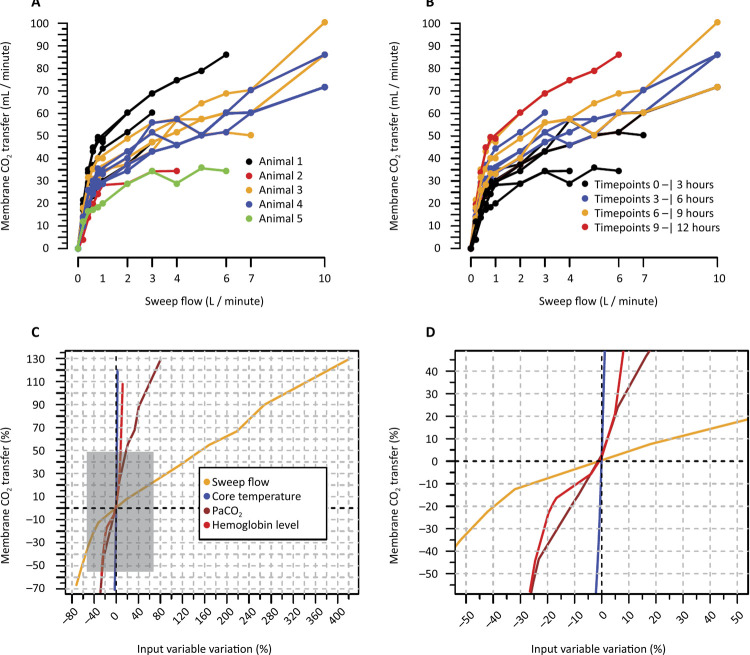
Carbon dioxide transfer across the oxygenation membrane according to the transfer-related variables. CO2 - carbon dioxide; PCO_2_ - partial pressure of carbon dioxide.

Multipaneled figure 1 illustrates the relationship between the SGF and CO_2_ transfer. Panel A demonstrates the expected increase in CO_2_ transfer as the SGF increases, emphasizing the association for each animal. Panel B focuses on each timepoint interval, with later time points demonstrating greater CO_2_ transfer. Finally, Panel C presents a spider plot of the unadjusted associations between other factors and CO_2_ transfer, with Panel D providing a magnified view of the near-zero coordinates from Panel C.

Panel A: carbon dioxide transfer according to the sweep gas flow titration in each animal at each timepoint where the sweep gas flow titrations were performed (individual colors show the analyzed animal). Panel B: Carbon dioxide transfer of each sweep gas flow titration according to the time point of the study (individual colors show three-hour time points). Panel C shows the spider plot of the graphic association of the percent variation in each multivariate selected variable (see the main manuscript) with the percent variation in carbon dioxide transfer. Panel D shows the magnification of the gray area in Panel C.

## DISCUSSION

Our results indicate that CO_2_ transfer using a 0.4m^2^ oxygenation membrane in a low-blood-flow CVVHD system can achieve transfer rates as high as 80 - 90mL/minute. A higher SGF, temperature, PaCO_2_, and hemoglobin level and a lower SaO_2_ were associated with higher CO_2_ transfer rates. The clinical importance of each of these variables depends on their potential for bedside manipulation within feasible physiological ranges.

The high diffusibility of CO_2_ enhances its convection capacity through the membrane, making SGF a crucial adjustable variable in low-flow CO_2_ removal;^(1,3,7,8)^however, the low range of the other independent variable variations precludes us from determining the real importance of each variable’s impact on CO_2_ transfer. Additionally, with a high bicarbonate concentration in the fluid delivered before the oxygenation membrane (in the dialysis filter), we expect a local increase in CO_2_ production, ultimately resulting in an increase in the preoxygenation membrane CO_2_ partial pressure and increased CO_2_ transfer.^(12)^

Elevated hemoglobin levels facilitate improved CO_2_ binding and transport, and a lower SaO_2_ is associated with greater CO_2_ transfer.^(13)^ Additionally, higher temperatures may increase the systemic metabolic rate and CO_2_ production (VCO_2_), contributing to greater CO_2_ transfer, although the temperature effect on carbonic anhydrase is minimal within physiological limits.^(14)^Hemoglobin could be more easily increased to enhance CO_2_ transfer (3.5mL/minute per g/dL increase in hemoglobin), while the effects of SaO_2_ would be negligible within usual ranges of saturation, and temperature manipulation to enhance CO_2_ transfer is not usually desirable.

Importantly, increasing PaCO_2_ is a second key modulator of increased CO_2_ transfer. In this experiment, higher PaCO_2_ levels occurred over time as hypoventilation ensued and arterial bicarbonate levels increased due to the high bicarbonate dialysate. The high bicarbonate concentration in the dialysate, which massively increased the concentration of CO_2_ due to mass conservation, could partially explain the high CO_2_ transfer;^(9)^ however, PaCO_2_, a surrogate of the premembrane PCO_2_, is still related to CO_2_ transfer, despite the very low CO_2_ mass. This combination of the high bicarbonate dialysate in series with CO_2_ removal may be key to improving CO_2_ transfer.

This study has limitations: first, it was not designed for this specific purpose. Second, the sample was small, although the results were consistent within animals. Third, despite the use of a mixed model, there are asymmetrical instances of SGFt between animals; fourth, the variation in SGF during SGFt could modify the premembrane PCO_2,_ leading to a carry-over phenomenon; however, the arterial PCO_2_ kinetics in low-flow systems are much slower.^(15)^ Fifth, during decarboxylation, the cardiac output is an important variable^(10)^ and a modulator of the arterial PaCO_2_, but not of the CO_2_ transfer after equilibrium.^(12)^ Sixth, we did not measure the after-membrane pH, which can be associated with hemolysis; and seventh, SGF was the only independent variable titrated during the experiment.

## CONCLUSION

In this study, we reaffirmed the importance of sweep gas flow in low-flow carbon dioxide removal during high-bicarbonate continuous veno-venous hemodialysis. Other carbon dioxide transfer modulating variables included the hemoglobin level, arterial oxygen saturation, partial pressure of carbon dioxide and core temperature. These results should be interpreted as exploratory to inform other well-designed experimental or clinical studies.
