# Supplementary material for: Factors associated with carbon dioxide transfer in an experimental model of severe acute kidney injury and hypoventilation during high bicarbonate continuous renal replacement therapy and oxygenation membrane support
Source: Crit Care Sci. 2024 Jun 19;36:e20240005en. doi: 10.62675/2965-2774.20240005-en (PMC11208041; doi:10.62675/2965-2774.20240005-en)
Supplement: Translation

# Translation: Fatores associados à transferência de dióxido de carbono em um modelo experimental de lesão renal aguda grave e hipoventilação durante terapia de substituição renal contínua com alto teor de bicarbonato e suporte com membrana de oxigenação

**DOI:** 10.62675/2965-2774.20240005-pt

**Authors:** Yuri de Albuquerque Pessoa dos Santos, Luis Carlos Maia Cardozo, Pedro Vitale Mendes, Bruno Adler Maccagnan Pinheiro Besen, Marcelo Park

**Affiliations:** 1 Brasil Unidade de Terapia Intensiva Médica, Hospital das Clínicas, Faculdade de Medicina, Universidade de São Paulo - São Paulo (SP), Brasil.

**Keywords:** Dióxido de carbono, Bicarbonatos, Insuficiência respiratória, Injúria renal aguda, Terapia de substituição renal, Animais

## Abstract

**Objetivo:**

Investigar os fatores que influenciam a transferência de dióxido de carbono em um sistema que integra uma membrana de oxigenação em série com terapia de substituição renal contínua com alto teor de bicarbonato em animais hipercápnicos.

**Métodos:**

Em um ambiente experimental, induzimos lesão renal aguda grave e hipercapnia em cinco porcos *Landrace* fêmeas. Em seguida, iniciamos terapia de substituição renal contínua com alto teor de bicarbonato (40mEq/L) com uma membrana de oxigenação em série para manter o pH acima de 7,25. Em intervalos de 1 hora, 6 horas e 12 horas após o início da terapia de substituição renal contínua, realizamos uma titulação padronizada do fluxo de gás de varredura para quantificar a transferência de dióxido de carbono. Avaliamos os fatores associados à transferência de dióxido de carbono através da membrana pulmonar com um modelo linear misto.

**Resultados:**

Realizamos 20 procedimentos de titulação do fluxo de gás de varredura, produzindo 84 medições de transferência de dióxido de carbono. A análise multivariada revelou associações entre os seguintes itens (coeficientes ± erros padrão): temperatura central (+7,8 ± 1,6 °C, p < 0,001), pressão parcial pré-membrana de dióxido de carbono (+0,2 ± 0,1mmHg, p < 0,001), nível de hemoglobina (+3,5 ± 0,6g/dL, p < 0,001), fluxo de gás de varredura (+6,2 ± 0,2L/minuto, p < 0,001) e saturação de oxigênio (-0,5% ± 0,2%, p = 0,019). Entre essas variáveis, e dentro das faixas fisiológicas avaliadas, o fluxo do gás de varredura foi o principal fator modificável que influenciou a eficácia da remoção de dióxido de carbono de baixo fluxo sanguíneo.

**Conclusão:**

O fluxo do gás de varredura é a principal variável relacionada à remoção de dióxido de carbono durante a terapia de substituição renal contínua com um alto nível de bicarbonato acoplado a um oxigenador. Outras variáveis moduladoras da transferência de dióxido de carbono incluíram o nível de hemoglobina, a saturação de oxigênio, a pressão parcial de dióxido de carbono e a temperatura central. Esses resultados devem ser interpretados como exploratórios para informar outros estudos experimentais ou clínicos bem planejados.

## INTRODUÇÃO

Os circuitos extracorpóreos de baixo fluxo são eficazes na remoção de dióxido de carbono (CO_2_), devido à sua alta difusibilidade de CO_2_.^(1)^ Esses sistemas têm sido empregados como terapias de resgate em ambientes clínicos.^(2)^ No entanto, o uso de membranas de oxigenação biocompatíveis menores (< 0,8m^2^) é insuficiente para corrigir adequadamente a acidose respiratória grave.^(3,4)^ Por outro lado, dialisatos com alto teor de bicarbonato (40mEq/L) em terapia de substituição renal contínua (TSRC) melhoram o controle do pH em modelos de bancada de lesão renal aguda hipercápnica.^(5)^ A combinação de membranas de oxigenação de superfície menor em série e TSRC com alto teor de bicarbonato pode ser uma intervenção em pacientes com insuficiência respiratória e lesão renal aguda, embora sua eficácia tenha sido pouco explorada em estudos de bancada.

Nosso objetivo foi investigar os fatores que influenciam a transferência de CO_2_ em um sistema que integra uma membrana de oxigenação em série e TSRC com alto teor de bicarbonato em animais hipercápnicos.

## MÉTODOS

Trata-se de análise secundária planejada de um experimento realizado na Faculdade de Medicina da Universidade de São Paulo, aprovada pelo Comitê de Ética no Uso de Animais (CEUA-17699/2022). No momento da publicação deste manuscrito, os resultados do estudo primário não tinham sido publicados.

### Instrumentação

O estudo priorizou o bem-estar animal, sendo os animais anestesiados e instrumentados conforme já descrito.^(5)^ Após a anestesia, posicionamos uma linha venosa central, um cateter 12F de 16cm de diálise venosa (Arrow™, PA, Estrados Unidos), um cateter Swan-Ganz (Edwards Lifesciences, Irvine, Estrados Unidos) e uma linha arterial. Realizou-se uma laparotomia mediana seguida de uma cistostomia para confirmar a anúria, e o hilo renal foi ligado em bloco. Os animais foram estabilizados por 1 hora após a cirurgia.

### Protocolo de hipercapnia

Após a estabilização, coletamos dados basais e induzimos a hipercapnia reduzindo o volume corrente para dois terços e ajustando a frequência respiratória para 40mrm. Após 1 hora, iniciamos a TSRC em série com um oxigenador. Nas 12 horas seguintes, ajustamos o volume corrente de hora em hora para atingir um pH arterial > 7,25, visando a um volume corrente mínimo de 3,5mL/kg. Durante esse período, foi mantido o suporte extracorpóreo, e os dados clínicos e laboratoriais foram coletados a cada hora.

### Suporte metabólico e respiratório extracorpóreo

Usamos um filtro de diálise Fx80 (Fresenius Kabi LTDA) com 30mL/kg de dialisato e uma taxa de fluxo sanguíneo de 3 a 4mL/kg/minuto. A heparina do filtro pré-diálise foi administrada em bólus de 15 a 20UI/kg, seguida de uma infusão horária na mesma taxa. A composição do dialisato sem fosfato foi [Na+] = 140,05mEq/L, [Cl-] = 103,85mEq/L, [K+] = 3,81mEq/L e [HCO3-] = 40,02mEq/L. O dialisato com alto teor de bicarbonato teve como objetivo otimizar o componente metabólico do pH^(5)^ para permitir uma redução mais rápida do volume corrente quando combinado com o efeito de descarboxilação do oxigenador.

A descarboxilação foi feita com o oxigenador Biocube2000 (Nipro Medical Ltda), que possui uma superfície de troca de 0,4m^2^ de fibras de polimetilpenteno. O fluxo de gás de varredura (SGF- *sweep gas flow*) foi mantido em 10L/minuto usando apenas oxigênio (FdO_2_ = 100%).

### Medição da transferência de dióxido de carbono

Quantificamos a transferência de CO_2_ estimando a pressão parcial de CO_2_ e o volume de gás exalado da saída do oxigenador, garantindo que não houvesse vazamento de gás. A pressão parcial de CO_2_ foi estimada usando um sensor infravermelho de CO_2_ expirado (E_T_CO_2_) integrado ao monitor multiparamétrico DX 2020 (Dixtal Ltda, São Paulo, Brasil).

O volume de gás exalado por minuto foi medido com um medidor de fluxo ajustável de precisão micrométrica conectado a um misturador de oxigênio e ar Sechrist 3500 (Sechrist Industries INC, Anaheim, CA, Estados Unidos).

A transferência de CO_2_ foi definida como a proporção de gás exalado por minuto, incluindo a pressão parcial de CO_2_ medida, estimada da seguinte forma: transferência de CO_2_ = (E_T_CO_2_/pressão barométrica) × (volume de gás/minuto). Os resultados são expressos em mL/minuto, considerando a pressão barométrica média de 700mmHg em São Paulo. Essa metodologia é consistente com as técnicas empregadas por Theodor Kolobow^(6)^ e foi mais refinada e testada por nosso grupo de pesquisa.^(7)^

### Protocolo de titulação do fluxo de gás de varredura

Realizou-se a titulação do SGF (SGFt) usando níveis predefinidos de SGF que variavam de 0 a 10L/minuto, um fluxômetro de precisão de oxigênio micro/macrométrico e um regulador de fluxo (Prevtech, São Paulo, SP, Brasil). Para cada medição do SGF, o fluxo foi reduzido de um valor inicial de 10L/minuto para o nível especificado. Observamos a curva e o valor do E_T_CO_2_ até a estabilização por 10 segundos, momento em que o E_T_CO_2_ foi registrado como a pressão parcial de CO_2_ exalado em equilíbrio naquele SGF. Nos casos em que o E_T_CO_2_ não era detectável em um determinado SGF, considerou-se a pressão parcial de CO_2_ anterior como o ponto mais baixo e a transferência de CO_2_ como o platô.

A SGFt foi predefinida e realizada em 1, 6 e 12 horas nos animais, com medições adicionais realizadas conforme necessário.

### Análise estatística

Os dados clínicos são apresentados como medianas [IQ25 - 75%]. As associações de SGF e a transferência de CO_2_ com outros possíveis fatores de influência são apresentadas em diagramas de espaguete e gráficos de aranha, respectivamente. Usando modelos lineares mistos com cada animal como um fator aleatório para considerar as observações agrupadas, analisamos as medições ao longo do tempo e a associação multivariável de possíveis fatores independentes com a transferência de CO_2_, empregando a eliminação retroativa para essa última. Esses fatores, extraídos de literatura prévia,^(6-10)^ incluíram pressão parcial de CO_2_ (PCO_2_) pré-membrana, níveis de hemoglobina, saturação de oxigênio (SaO_2_), SGF e temperatura central,^(6-10)^ com a PaCO_2_ servindo como substituta para a PCO_2_ pré-membrana. O fluxo sanguíneo, um fator independente em estudos de oxigenação por membrana extracorpórea (ECMO) com variações de fluxo mais altas,^(7,9)^ foi excluído da análise multivariável da TSRC devido às baixas taxas de fluxo. A análise estatística foi realizada com o programa R.^(11)^

## RESULTADOS

Incluímos cinco animais com peso médio de 33,1kg (28,7 - 35,0kg) e realizamos 20 procedimentos de SGFt e 84 medições de transferência de CO_2_. As características hemodinâmicas, respiratórias e metabólicas anteriores à SGFt estão detalhadas na tabela 1. O volume corrente diminuiu com um aumento significativo na PaCO_2_, embora o pH tenha permanecido acima de 7,25.

**Tabela 1:** Variáveis fisiológicas respiratórias, hemodinâmicas e metabólicas imediatamente antes da titulação do fluxo de gás de varredura da membrana

Intervalos de tempo	Basal	1 hora	2 horas	3 horas	4 horas	6 horas	7 horas	12 horas	Valor de p*
VCO_2_†/amostra de animais	84/5	84/5	37/5	38/5	12/5	38/5	42/5	25/5	
Variáveis respiratórias									
Volume corrente (mL)	300 [260,320]	200 [200,220]	180 [180,220]	170 [160,200]	160 [160,160]	150 [140,160]	140 [120,180]	120 [120,180]	< 0,010
Frequência respiratória (mrm)	35 [25,38]	40 [25,40]	40 [25,40]	40 [25,40]	40 [40,40]	40 [40,40]	40 [40,40]	40 [40,40]	< 0,010
FiO_2_ (%)	21 [21,21]	25 [25,31]	25 [21,28]	30 [25,35]	25 [25,25]	30 [25,30]	35 [35,40]	38 [30,38]	< 0,010
E_T_CO_2_ (mmHg)	36 [30,37]	38,50 [36,46]	43 [39,56]	47 [38,67]	43 [43,43]	47 [45,49]	62 [41,64]	87 [42,87]	< 0,010
Variáveis hemodinâmicas									
Débito cardíaco (L/minuto)	3,0 [2,6,3,6]	3,3 [2,4,3,4]	3,1 [3,0,3,5]	3,3 [2,5,3,5]	3,4 [3,4,3,4]	4,3 [3,1,5,6]	4,5 [4,0,8,4]	4,9 [4,0,4,9]	< 0,001
Frequência cardíaca (bpm)	111 [80,159]	157 [84,157]	129 [93,146]	137 [87,195]	132 [132,132]	157 [140,187]	154 [121,209]	129 [129,196]	< 0,010
PAPm (mmHg)	26 [25,35]	27 [25,28]	28 [26,28]	26 [23,27]	27 [27,27]	30 [29,35]	27 [27,32]	28 [28,30]	< 0,010
PAm (mmHg)	121 [101,123]	101,50 [80,117]	91 [88,94]	84 [70,86]	89 [89,89]	84 [71,90]	78 [75,105]	71 [41,71]	< 0,010
PVC (mmHg)	8 [7,10]	5 [5,9]	5 [3,7]	6 [3,7]	7 [7,7]	6 [3,7]	8 [7,9]	8 [8,8]	< 0,010
PAOP (mmHg)	12 [9,15]	12 [9,12]	8 [7,10]	9 [6,12]	10 [10,10]	9 [7,9]	9 [9,12]	8 [8,10]	< 0,010
Variáveis metabólicas e de TSRC									
Temperatura central (°C)	38,5 [38,3,39,1]	37,8 [37,1,37,8]	37,7 [37,1,38,2]	38,2 [37,0,38,4]	38,4 [38,4,38,4]	37,8 [37,0,38,7]	38,1 [37,8,39,0]	38,0 [37,6,38,0]	< 0,001
Fluxo sanguíneo (mL/minuto)	187,50 [96,202]	187,50 [96,204]	167 [96,205]	172 [95,182]	208 [208,208]	178 [96,205]	182 [96,198]	96 [96,210]	< 0,010
Nível de hemoglobina (g/dL)	9,8 [7,8,11,0]	10,0 [8,4,11,0]	10,2 [7,5,11,1]	10,2 [7,5,11,1]	7,7 [7,7,7,7]	11,0 [10,7,11,3]	8,5 [8,2,11,4]	11,1 [7,5,11,1]	< 0,001
pH	7,44 [7,41,7,49]	7,36 [7,31,7,42]	7,39 [7,30,7,42]	7,39 [7,30,7,42]	7,36 [7,36,7,36]	7,32 [7,31,7,37]	7,26 [7,22,7,33]	7,25 [7,25,7,32]	< 0,001
PaCO_2_ (mmHg)	31 [30,37,90]	36 [35,48,15]	43 [37,58,40]	43 [37,58,40]	44 [44,44]	50 [50,51]	64,20 [56,67,90]	86,70 [43,86,70]	< 0,010
PaO_2_ (mmHg)	75,90 [70,86]	76,47 [73,95,85]	75 [72,76]	75 [72,76]	77 [77,77]	83 [63,91]	89,30 [73,91]	80,90 [80,90,92]	< 0,010
Saturação de oxigênio (%)	97 [93,20,98]	95,50 [90,55,99,15]	93 [87,90,94,50]	93 [87,90,94,50]	93,80 [93,80,93,80]	95 [94,60,96,20]	88 [84,89,30]	90,30 [90,30,95]	< 0,010
SBE (mEq/L)	-0,2 [-1,4,0,2]	1,1 [-0,8,3,9]	1,6 [0,1,2,4]	1,6 [0,1,2,4]	0,3 [0,3,0,3]	-0,2 [-4,6,4,2]	0,1 [-1,7,7,4]	9,3 [-3,3,9,3]	< 0,001

VCO_2_ - produção de dióxido de carbono; mrm - movimentos respiratórios por minuto; FiO_2_ - fração inspiratória de oxigênio; E_T_CO_2_ - pressão parcial de dióxido de carbono expirado; bpm - batimentos por minuto; PAPm - pressão arterial pulmonar média; PAm - pressão arterial média sistêmica; PVC - pressão venosa central; PAOP - pressão arterial de oclusão pulmonar; TSRC - terapia de substituição renal contínua; PCO_2_ - pressão parcial de dióxido de carbono; PaO_2_ - pressão parcial de oxigênio; SBE - excesso de base padrão. * Valor de p extraído da evolução temporal de cada variável, usando um modelo linear misto de interação fator *versus* tempo com o animal individual como fator aleatório; † Aqui, a produção de dióxido de carbono é o número de sequências de titulação de fluxo de gás de varredura.

A análise multivariável produziu os seguintes resultados [coeficiente ± erro padrão (valor de p)]: um intercepto = -271,6 ± 63,4 (p < 0,001), uma temperatura central (ºC) = +7,8 ± 1,6 (p < 0,001), PCO_2_ pré-membrana (mmHg) = +0,2 ± 0,1 (p < 0,001), nível de hemoglobina (g/dL) = +3,5 ± 0,6 (p < 0,001), SaO_2_ (%) = -0,5 ± 0,2 (p = 0,019) e SGF (L/minuto) = +6,2 ± 0,2 (p < 0,001).

A figura 1, com vários painéis, ilustra a relação entre o SGF e a transferência de CO_2_. O painel A demonstra o aumento esperado na transferência de CO_2_ à medida que o SGF aumenta, enfatizando a associação entre cada animal. O painel B concentra-se em cada intervalo de tempo, e os intervalos posteriores demonstram maior transferência de CO_2_. Por fim, o painel C apresenta um gráfico de aranha das associações não ajustadas entre outros fatores e a transferência de CO_2_, enquanto o painel D fornece uma visão ampliada das coordenadas quase nulas do painel C.

**Figura 1:** Transferência de dióxido de carbono através da membrana de oxigenação, conforme as variáveis relacionadas à transferência.

Painel A: transferência de dióxido de carbono conforme a titulação de fluxo de gás de varredura em cada animal em cada momento de realização das titulações de fluxo de gás de varredura (as cores individuais mostram o animal analisado). Painel B: transferência de dióxido de carbono de cada titulação de fluxo de gás de varredura conforme o momento do estudo (as cores individuais mostram os intervalos de tempo de 3 horas). O painel C mostra o gráfico de aranha da associação gráfica da variação percentual em cada variável multivariada selecionada (consulte o manuscrito principal) com a variação percentual na transferência de dióxido de carbono. O painel D mostra a ampliação da área cinza no painel C.

CO_2_ - dióxido de carbono; PCO_2_ - pressão parcial de dióxido de carbono.

## DISCUSSÃO

Nossos resultados indicam que a transferência de CO_2_ usando uma membrana de oxigenação de 0,4m^2^ em um sistema TSRC de baixo fluxo sanguíneo pode atingir taxas de transferência de 80 a 90mL/minuto. Níveis mais altos de SGF, temperatura, PaCO_2_, hemoglobina e SaO_2_ mais baixa foram associados a taxas mais altas de transferência de CO_2_. A importância clínica de cada uma dessas variáveis depende de seu poder de manipulação no leito dentro de faixas fisiológicas viáveis.

A alta difusibilidade do CO_2_ aumenta sua capacidade de convecção através da membrana, tornando o SGF uma variável ajustável crucial na remoção de CO_2_ de baixo fluxo.^(1,3,7,8)^ Contudo, o baixo intervalo das variações das outras variáveis independentes nos impede de determinar a real importância do impacto de cada variável na transferência de CO_2_. Além disso, com uma alta concentração de bicarbonato no fluido administrado antes da membrana de oxigenação (no filtro de diálise), esperamos um aumento local na produção de CO_2_, resultando, em última análise, em um aumento na pressão parcial de CO_2_ da membrana de pré-oxigenação e em uma maior transferência de CO_2_.^(12)^

Níveis elevados de hemoglobina facilitam uma melhor ligação e transporte de CO_2_, e uma SaO_2_ mais baixa está associada a uma maior transferência de CO_2_.^(13)^ Além disso, temperaturas mais altas podem aumentar a taxa metabólica sistêmica e a produção de CO_2_ (VCO_2_), contribuindo para uma maior transferência de CO_2_, embora o efeito da temperatura sobre a anidrase carbônica seja mínimo dentro dos limites fisiológicos.^(14)^ A hemoglobina poderia ser aumentada mais facilmente para aumentar a transferência de CO_2_ (3,5mL/minuto por aumento de g/dL na hemoglobina), enquanto os efeitos da SaO_2_ seriam insignificantes dentro das faixas usuais de saturação, não sendo geralmente desejável a manipulação da temperatura para aumentar a transferência de CO_2_.

É importante ressaltar que o aumento da PaCO_2_ é um segundo modulador importante do aumento da transferência de CO_2_. Nesse experimento, os níveis mais altos de PaCO_2_ ocorreram com o passar do tempo, à medida que a hipoventilação se instalou e os níveis de bicarbonato arterial aumentaram devido ao dialisato com alto teor de bicarbonato. A alta concentração de bicarbonato no dialisato, que aumentou maciçamente a concentração de CO_2_ devido à conservação da massa, poderia explicar parcialmente a alta transferência de CO_2_.^(9)^ No entanto, a PaCO_2_, um substituto da PCO_2_ pré-membrana, ainda está relacionada à transferência de CO_2_, apesar da massa muito baixa de CO_2_. Essa combinação do dialisato com alto teor de bicarbonato em série e a remoção de CO_2_ pode ser a chave para melhorar a transferência de CO_2_.

Este estudo tem limitações. Primeiramente, ele não foi projetado para essa finalidade específica. Em segundo lugar, a amostra foi pequena, embora os resultados tenham sido consistentes entre os animais. Em terceiro lugar, apesar do uso de um modelo misto, há instâncias assimétricas de SGFt entre os animais. Em quarto lugar, a variação do SGF durante a SGFt poderia modificar a PCO_2_ pré-membrana, levando a um fenômeno de *carry-over*. No entanto, a cinética da PCO_2_ arterial em sistemas de baixo fluxo é muito mais lenta.^(15)^ Em quinto lugar, durante a descarboxilação, o débito cardíaco é uma variável importante^(10)^ e um modulador da PaCO_2_ arterial, mas não da transferência de CO_2_ após o equilíbrio.^(12)^ Em sexto lugar, não medimos o pH pós-membrana, que pode estar associado à hemólise. E, em sétimo lugar, o SGF foi a única variável independente titulada durante o experimento.

## CONCLUSÃO

Neste estudo, reafirmamos a importância do fluxo de gás de varredura na remoção de dióxido de carbono de baixo fluxo durante a terapia de substituição renal contínua com alto teor de bicarbonato. Outras variáveis moduladoras da transferência de dióxido de carbono incluíram o nível de hemoglobina, a saturação de oxigênio, a pressão parcial de dióxido de carbono e a temperatura central. Esses resultados devem ser interpretados como exploratórios para informar outros estudos experimentais ou clínicos bem planejados.
